# Alternative oxidase gene induced by nitric oxide is involved in the regulation of ROS and enhances the resistance of *Pleurotus ostreatus* to heat stress

**DOI:** 10.1186/s12934-021-01626-y

**Published:** 2021-07-19

**Authors:** Ludan Hou, Mengran Zhao, Chenyang Huang, Qi He, Lijiao Zhang, Jinxia Zhang

**Affiliations:** 1 Institute of Agricultural Resources and Regional Planning, Chinese Academy of Agricultural Sciences, 10081 Beijing, China; 2 Key Laboratory of Microbial Resources, Ministry of Agriculture and Rural Affairs, 10081 Beijing, China; 3 Jilin Agricultural University, 130118 Jilin, China

**Keywords:** *Pleurotus ostreatus*, Nitric oxide, ROS, RNA-Seq, Alternative oxidase, Antioxidant enzymes

## Abstract

**Background:**

In China, during the cultivation process of *Pleurotus ostreatus*, the yield and quality of fruiting bodies are easily affected by high temperatures in summer. Nitric oxide (NO) plays an important regulatory role in the response to abiotic stress, and previous studies have found that NO can induce alternative oxidase (*aox*) experssion in response to heat stress (HS) by regulating aconitase. However, the regulatory pathway of NO is complex, and the function and regulation of the *aox* gene in the response to HS remain unclear.

**Results:**

In this study, we found that NO affected nicotinamide adenine dinucleotide (NADH) and adenosine triphosphate (ATP) levels, reduced hydrogen peroxide (H_2_O_2_) and superoxide anion (O_2_^−^) contents, and slowed O_2_^−^ production. Further RNA-Seq results showed that NO regulated the oxidation-reduction process and oxidoreductase activity, affected the cellular respiration pathway and activated *aox* gene expression. The function of *aox* was determined by constructing overexpression (OE) and RNA interference (RNAi) strains. The results showed that the OE-*aox* strains exhibited obviously improved growth recovery after exposure to HS. During exposure to HS, the OE-*aox* strains exhibited reduced levels of NADH, the product of the tricarboxylic acid (TCA) cycle, and decreased synthesis of ATP, which reduced the production and accumulation of reactive oxygen species (ROS), whereas the RNAi-*aox* strains exhibited the opposite result. In addition, *aox* mediated the expression of antioxidant enzyme genes in the mycelia of *P. ostreatus* under HS through the retrograde signaling pathway.

**Conclusions:**

This study shows that the expression of the *aox* gene in *P. ostreatus* mycelia can be induced by NO under HS, that it regulates the TCA cycle and cell respiration to reduce the production of ROS, and that it can mediate the retrograde signaling pathway involved in the mycelial response to HS.

**Graphical abstract:**

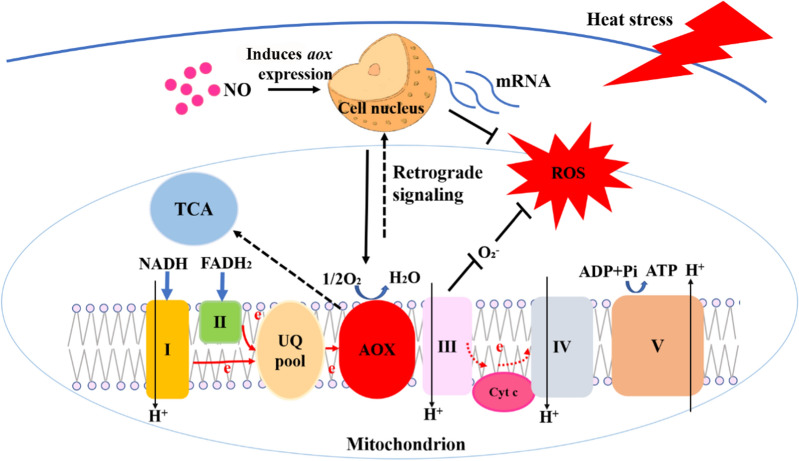

**Supplementary Information:**

The online version contains supplementary material available at 10.1186/s12934-021-01626-y.

## Background

*Pleurotus ostreatus* is one of the most widely cultivated mushroom species globally and is mainly cultivated in horticultural facilities in China [1]. During the cultivation process of *P. ostreatus*, high temperatures in summer limit the growth of mycelia, the formation of primordia and the development of fruiting bodies, and thereby seriously affecting the yield. However, the mycelia response to changes in temperature is very complex and includes different physiological and metabolic changes that affect the overall development of edible fungi as well as interactions between cells and molecules. Metabolomics analyses have shown that heat stress (HS) promotes the degradation of unsaturated fatty acids and nucleotides, increases the contents of amino acids and vitamins, and accelerates glycolysis and the tricarboxylic acid (TCA) cycle [2]. Furthermore, reactive oxygen species (ROS) accumulate in the mycelium during exposure to HS, which results in cell damage and even apoptosis [3]. The synthesis of trehalose and heat shock proteins is an important component of the response of edible fungi to high temperatures and to their resistance to heat [4]. Moreover, nitric oxide (NO) and calcium (Ca^2+^) as signaling molecules, also play an active protective role during exposure to HS [5]. In recent years, increasing attention has been given to the mechanism through which fungi respond to HS.

NO, an essential endogenous signaling molecule that is involved in many biological processes in plants, animals, bacteria, and fungi, is considered a broad-spectrum antistress molecule [6–8]. The role of NO as a signaling molecule in the growth, development and stress responses of plants has been widely studied and has become the focus of much research. Previous studies have revealed that NO contributes to various aspects of stress tolerances by influencing ROS metabolism [9]. Ca^2+^-mediated NO signal transduction is involved in the response of *Brassica* seedlings to metalloid stress [10], and in *Prunus persica* (L.) Batsch, treatment with a NO solution can protect the fruit from pathogen infection by inducing the activity of defense enzymes and the expression of pathogenesis-related genes [11]. In two ecotypes of reeds, NO significantly reduces the contents of hydrogen peroxide (H_2_O_2_) and malondialdehyde in calli and significantly inhibits the increase in ion leakage and growth inhibition and decrease in cell viability induced by HS [12]. Previous studies have shown that NO can regulate ROS content in two ways: by acting as a free radical to neutralize ROS or by acting as a signaling molecule to initiate gene expression via a molecular cascade [13]. In fungi, the function of NO has been gradually studied in recent years. For example, during fungal development, NO might not only induce sexual development [14] but it may also be involved in conidiation, spore germination, and formation of the parasitic appressoria structure, among other processes [15, 16]. In addition, studies have shown that NO can participate in the regulation of secondary metabolism in fungi. For example, NO can stimulate the activities of phenylalanine ammonia lyase and chalcone synthase in fruiting bodies to induce the accumulation of phenols and quinolines [17]. In addition, NO participates in the response pathway of fungi to various environmental stresses [18, 19]. Recently, NO has also been found in edible mushroom. Specifically, in *Ganoderma lucidum*, NO is involved in the regulation of ganoderic acid synthesis [5], and in *P. ostreatus*, NO can alleviate the oxidative damage induced in mycelia by HS [20]. However, the function and regulation of NO in fungi is less clear than in plants.

Mitochondria are the main site of ROS bursts and play a key role in cell energy metabolism. The electron transport chain (ETC) of mitochondria consists of two pathways: the main cytochrome c pathway and the alternative pathway. Alternative oxidase (AOX, encoded by *aox*), an integral protein (32–36 kDa) of the inner mitochondrial membrane [21], is a component of the mitochondrial respiratory pathway that is widely found in higher plants and some fungi and algae and is responsible for the activity of the alternative respiratory pathway [22]. In *Neurospora crassa*, a model fungal organism, *aox* transcripts are undetectable or present at very low levels under normal growth conditions [23]. However, when cells are grown in the presence of drugs that inhibit the cytochrome-mediated electron transport chain, such as antimycin A, which inhibits complex III, expression of the *aox* gene is strongly induced and the AOX protein can be found in mitochondria [24]. In addition, AOX is also present in mutant strains that lack components of the cytochrome-mediated electron transport chain [25]. Recently, the function of AOX has been widely studied. In plants, the AOX pathway plays an important role in maintaining metabolism and signal homeostasis during exposure to abiotic and biotic stress [26]. One of the possible functions of AOX is regulating the production of ROS [27, 28]. The electron transport flow generated by AOX bypasses proton pump complexes III and IV, which affects adenosine triphosphate (ATP) production and reduces electron leakage and ROS production [29]. In tobacco, AOX can maintain plant environmental homeostasis and enhance the tolerance of cells to drought stress by controlling the respiration rate, photosynthesis and chlorophyll synthesis [30]. In *Medicago truncatula*, NO can induce the expression of *aox*, and AOX can help regulate the accumulation of ROS, protect the photosystem, and enhance plant resistance to salt stress [31]. In addition, AOX enhances the tolerance of spring wheat to HS [32]. The function of AOX in fungi has also been reported. For instance, AOX is a determinant for growth and sporulation in the early diverging fungus *Blastocladiella emersonii* [33] and in *Aspergillus fumigatus*, silencing of the mitochondrial *aox* gene makes the strain more sensitive to ROS, and easier to kill by macrophages [34]. However, the function of AOX in *P. ostreatus* remains unclear.

The expression of the *aox* gene is also induced by a wide range of biotic and abiotic stresses [35], and previous studies have shown that upreglation of the *aox* gene under stress conditions is associated with stress-dependent ROS production in tobacco [36]. Our previous study revealed that NO, as a signaling molecule under HS, can increase the citric acid content by inhibiting aconitase in *P. ostreatus.* The accumulation of citric acid can induce the expression of the *aox* gene and enhance the resistance of mycelia to HS [20]. However, the regulatory pathway through which NO alleviates mycelial stress is complex, and the mechanism through which the *aox* gene of *P. ostreatus* enhances the thermostability of mycelia remains unclear; hence, further research is needed to obtain a more in-depth understanding of this phenomenon. In this study, RNA sequencing (RNA-Seq) technology was used to explore the possible regulatory pathways and key genes involved in the NO-mediated alleviation of HS-induced damage in *P. ostreatus*, and the regulatory effect of NO on the expression of the *aox* gene was studied in detail. Moreover, the relationships among *aox*, energy metabolism and ROS were explored using overexpression (OE) and RNA interference (RNAi) technology.

## Results

### Exogenous NO affected the energy metabolism of mycelia and reduced the production and accumulation of ROS

**Fig. 1:**
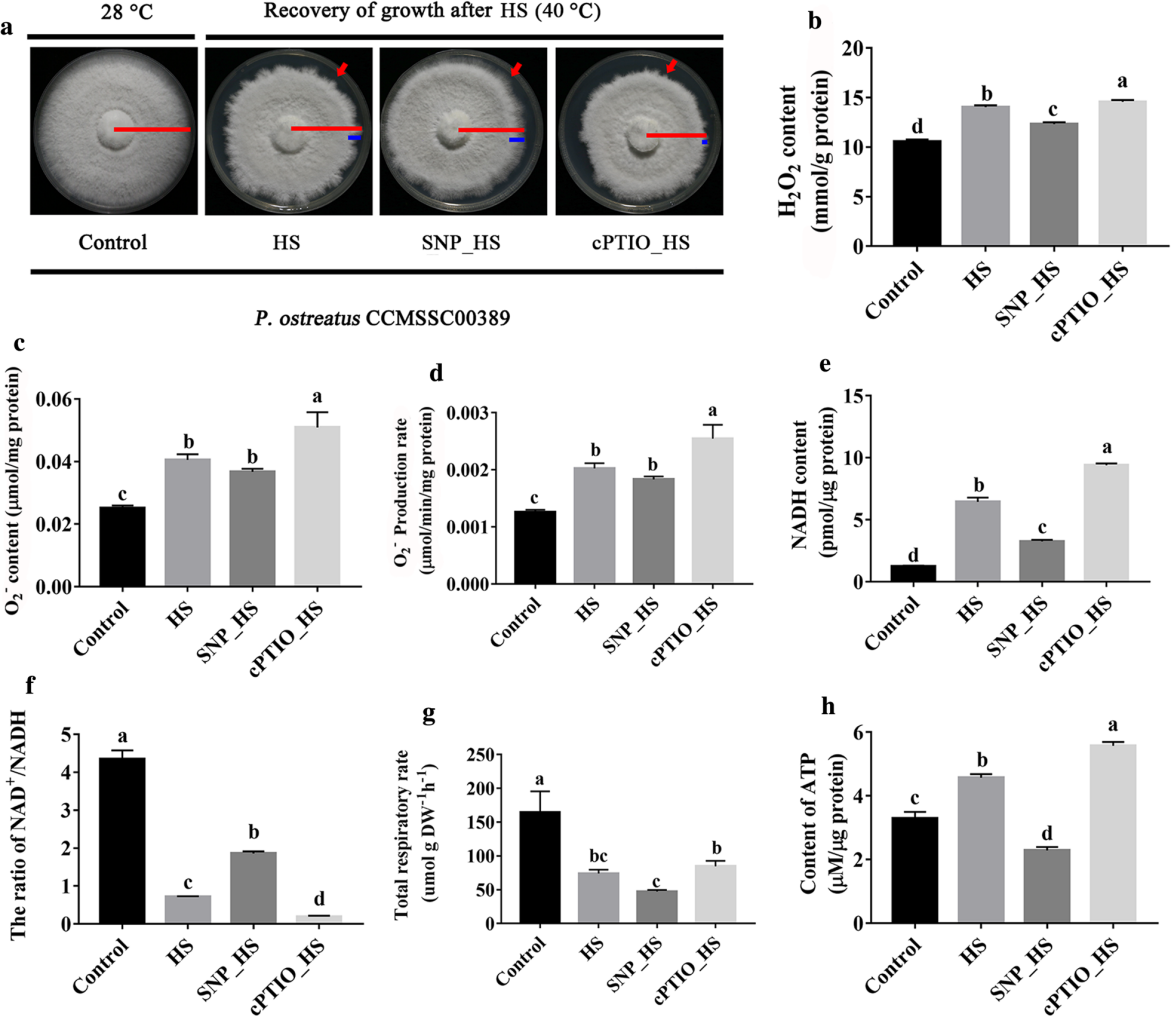
The addition of exogenous NO reduced the degree of mitochondrial damage and the accumulation of ROS in mycelia under HS. **A** Effect of exogenous NO on mycelial growth after HS. The red lines represent the radius of mycelial growth throughout the growth period, and the blue lines represent the radius of mycelial growth during recovery from HS (40 °C for 48 h). The red arrows point to mycelia that experienced growth recovery. **B** Effect of exogenous NO on the H_2_O_2_ content in mycelia after HS. **C** O_2_^−^ content. **D** O_2_^−^ production rate. E.NADH content. **F** NAD^+^/NADH ratio. **G** Total respiratory rate. **H** ATP content. The values are presented as the means ± SEs from three independent experiments. Different letters indicate significant differences among the samples (*P* < 0.05 according to Duncan’s test)

NO, an important signaling molecule, plays an important regulatory role in the growth and development of organisms and their responses to stress. Our previous study showed that NO plays an important role in the response of *P. ostreatus* to HS [20]. In this study, the addition of exogenous NO promoted the recovery of *P. ostreatus* mycelial growth after HS, as shown in Fig. 1A (the red arrow indicates regenerated mycelia after HS). H_2_O_2_ and O_2_^−^ are important components of ROS, and as shown in Fig. 1B, the content of H_2_O_2_ in mycelia increased significantly after HS (HS vs. control, *P* = 1.118 × 10^− 6^). Exogenous sodium nitroprusside (SNP, 100 µM) significantly reduced the accumulation of H_2_O_2_ in mycelia after HS (SNP_HS vs. HS, *P* = 2.430 × 10^− 4^), but the level was still significantly higher than that in the control group (SNP_HS vs. control, *P =* 1.864 × 10^− 4^). As shown in Figs. 1 C and D, HS increased the O_2_^−^ content in mycelia by 1.64-fold and the production rate of O_2_^−^ by 60.74 %. In addition, compared with the levels found in the HS group, exogenous SNP decreased the O_2_^−^ content and production rate by 12.85 and 9.34 %, respectively. 2-(4-carboxyphenyl)-4,4,5,5-tetramethylimidazoline-1-oxyl3-oxidec (cPTIO) is an NO scavenger. Research shows that the addition of 250 µM cPTIO can significantly increase the content and production rate of O_2_^−^. Our results showed that exogenous NO could affect not only the content of H_2_O_2_, but also the content and production rate of O_2_^−^.

Nicotinamide adenine dinucleotide (NAD) is a coenzyme that exists in all cells and is found in two forms: oxidized (NAD^+^) and reduced (NADH). NADH is produced during glycolysis pathway and the TCA cycle, participates in material and energy metabolism in cells and serves as a control marker in the energy production chain of mitochondria. As shown in Fig. 1E, HS significantly increased the content of NADH in mycelia compared with the level found in the control group (HS vs. control, *P* = 1.500 × 10^− 9^), and this increase could be partially offset by treatment with SNP. HS also significantly affected the NAD^+^/NADH ratio (HS vs. control, *P* = 3.429 × 10^− 9^), and the addition of exogenous SNP alleviated the imbalance in NAD caused by HS. In contrast, the addition of cPTIO significantly decreased the NAD^+^/NADH ratio compared with that observed in the HS group (cPTIO_HS vs. HS, *P* = 0.007) (Fig. 1F). Because most NAD^+^ is reduced to NADH by the TCA cycle in mitochondria and NADH serves as a substrate for the generation of ROS in the respiratory chain [37], it can be assumed that the TCA cycle is accelerated by HS and that NO can affect the accumulation of NADH by regulating the TCA cycle. ATP is commonly considered as intracellular energy currency molecule, and the mitochondrial respiratory chain is the site of ATP production. As shown in Fig. 1G, the total respiratory rate of mycelia decreased after HS, and this decrease might be caused by mitochondrial damage induced by HS. The addition of exogenous NO further inhibited the total respiratory rate. It has been suggested that NO can regulate the respiratoy rate of mycelia under stress and that NO might reduce ROS production by inhibiting the respiratory rate. Interestingly, the ATP content increased by 39.27 % after HS treatment compared with the level found in the control group, and this increase was synchronous with the inceases in O_2_^−^ and H_2_O_2_. The increase in the ATP content might be due to the observed increase in the NADH levels (Fig. 1E). Exogenous NO significantly inhibited the increase in ATP induced by HS(SNP_HS vs. HS, *P* = 2.237 × 10^− 6^), whereas the addition of exogenous cPTIO significantly increased the ATP content under HS (cPTIO_HS vs. HS, *P* = 0.001) (Fig. 1H). The results further suggested that NO can regulate the respiratory chain.

In conclusion, NO might alleviate the inceases in O_2_^−^ and H_2_O_2_ by regulating the mitochondrial respiratory chain and thereby reducing mycelial damage induced by HS.

### RNA-Seq analysis of the regulatory mechanism through which NO alleviates mycelial damage induced by HS

To fully understand the effect of NO on the transcriptome of *P. ostreatus* under HS, 12 RNA-Seq cDNA libraries were prepared. As shown in Additional file 1: Table S2, after removing adapters, low-quality regions and all possible contamination, each treatment group contained an average of 47.29 M clean reads with a quality score of 30 (Q30) > 94.93 % and a GC percentage between 53.03 and 53.64 %. The ratio of reads mapping to the *P. ostreatus* genome was high, ranging from 77.26 to 82.93 %. This result indicated that the accuracy of the sequencing results was high and could be used for subsequent analysis.

**Fig. 2:**
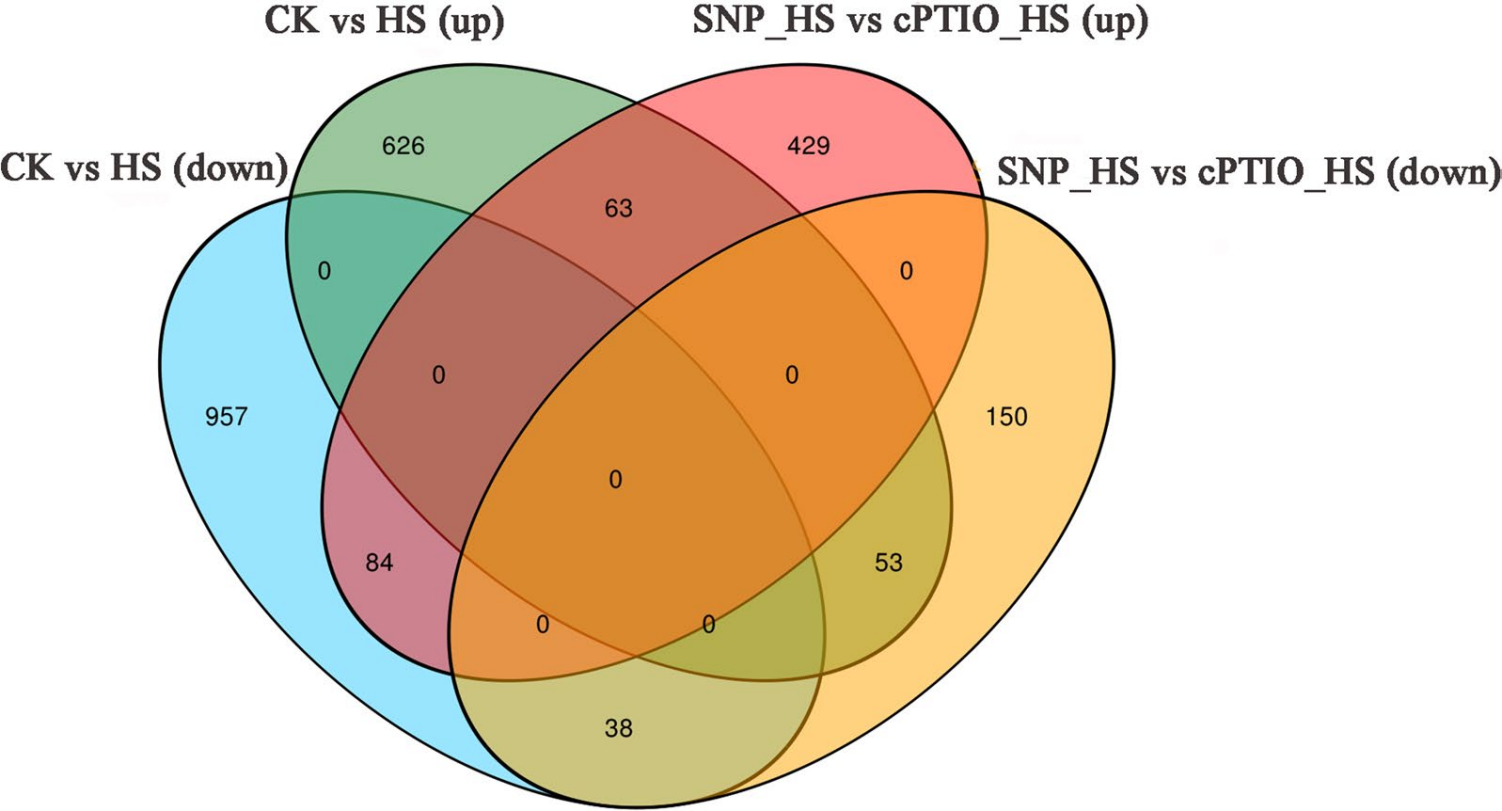
Number of up and downregulated genes among the CK, HS, SNP_HS and cPTIO_HS groups

**Fig. 3:**
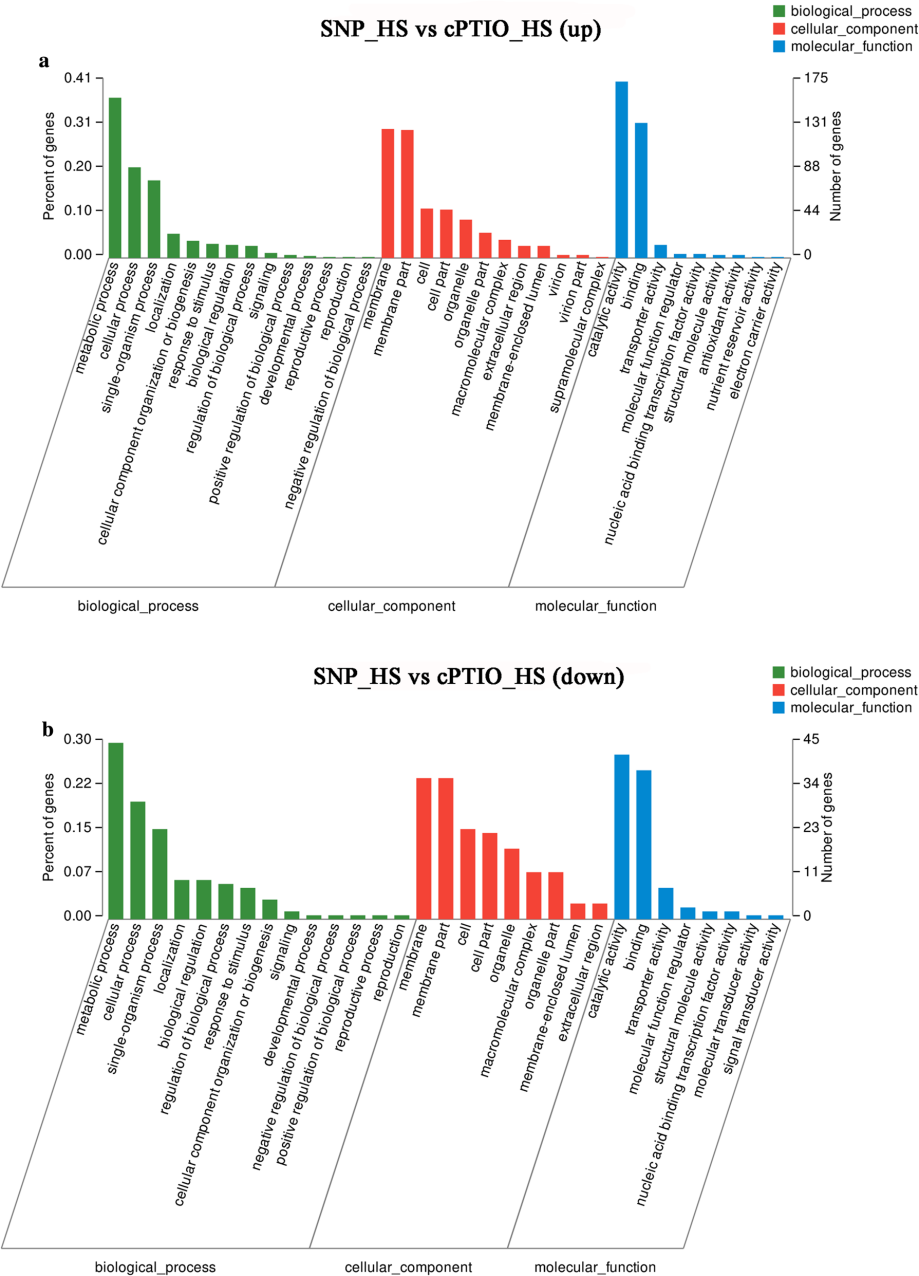
Functional classification of GO terms assigned to the DEGs. **A** Upregulated DEGs identified from the SNP_HS vs. cPTIO_HS comparison. **B** Downregulated DEGs identified from the SNP_HS vs. cPTIO_HS comparison. The y-axis on the left represents the percentage of DEGs assigned the GO term, and the y-axis on the right shows the number of DEGs

Based on the RNA-Seq analysis, the functions of 579 differentially expressed genes (DEGs) identified from the SNP_HS vs. cPTIO_HS comparison were examined to elucidate the possible mechanism through which NO alleviates the oxidative damage to mycelia induced by HS (Fig. 2). In the gene ontology (GO) analysis, the DEGs identified from the SNP_HS vs. cPTIO_HS comparison were classified into three categories: ‘biological process’, ‘cellular component’ and ‘molecular function’ (Fig. 3). Within the biological process category, genes corresponding to metabolic processes, cellular processes and single organism process were the most abundant. Within the cellular component category, the most abundant terms of DEGs were membrane and membrane parts, and within the molecular function category, catalytic activity and binding were the most abundant terms. These results indicated that NO might participate in the mycelial response to HS by regulating cell metabolism, affecting cell membrane components and structure, and affecting the catalytic activity of proteins.

**Fig. 4:**
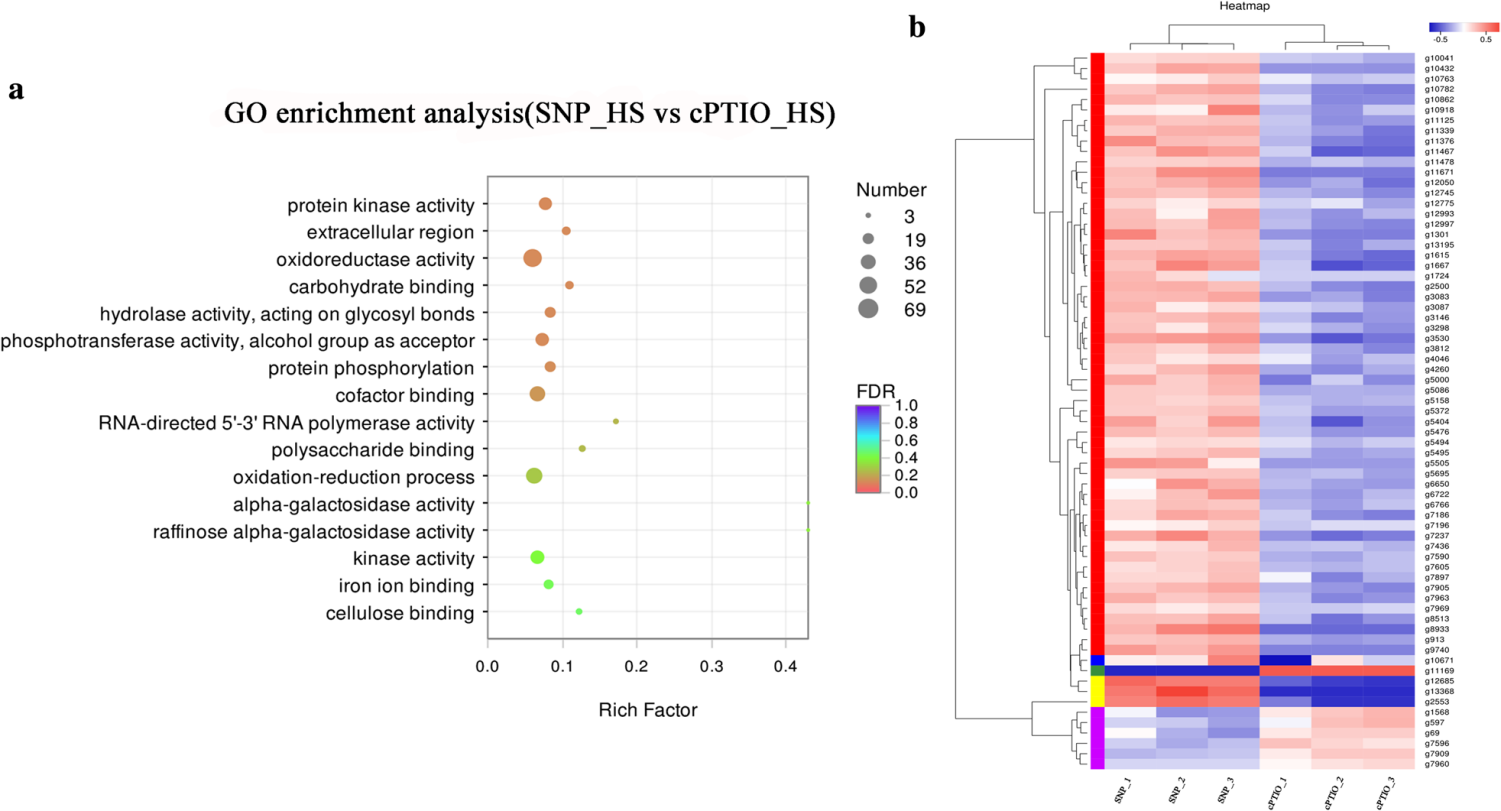
Significantly enriched pathways obtained for the DEGs and heatmap of oxidoreductase activity. **A** Significantly enriched pathways obtained for the DEGs identified from the SNP_HS vs. cPTIO_HS comparison. **B** Heatmap of the antioxidant system (oxidation-reduction process and oxidoreductase activity) consisting of the DEGs identified from the SNP_HS vs. cPTIO_HS comparison

To understand the functions of the DEGs identified from the SNP_HS vs. cPTIO_HS comparison, a pathway enrichment analysis was performed. The results showed that the DEGs were mainly concentrated in the following pathways: oxidoreductase activity, oxidation-reduction process, cofactor binding, protein kinase activity, phosphotransferase activity, alcohol group as acceptor, and protein phosphorylation (Fig. 4A). Previous studies have shown that HS can lead to the production and accumulation of ROS in mycelia, and ROS can further cause oxidative damage. As shown in Fig. 4A, exogenous NO can affect the oxidation-reduction process and oxidoreductase activity. In addition, considering the close relationship between the antioxidant system and ROS clearance, the expression pattern of the genes after the addition of an NO donor or scavenger was further analyzed with a heatmap. As shown in Fig. 4B, 69 DEGs identified after the addition of SNP or cPTIO were enriched in the oxidation-reduction process pathway and oxidoreductase activity, and these included 62 significantly upregulated DEGs and seven downregulated DEGs. It can thus be hypothesized that NO can activate the activity of oxidoreductase and accelerate redox reactions under HS.

### NO affected the expression of key genes in the respiratory chain under HS

**Fig. 5:**
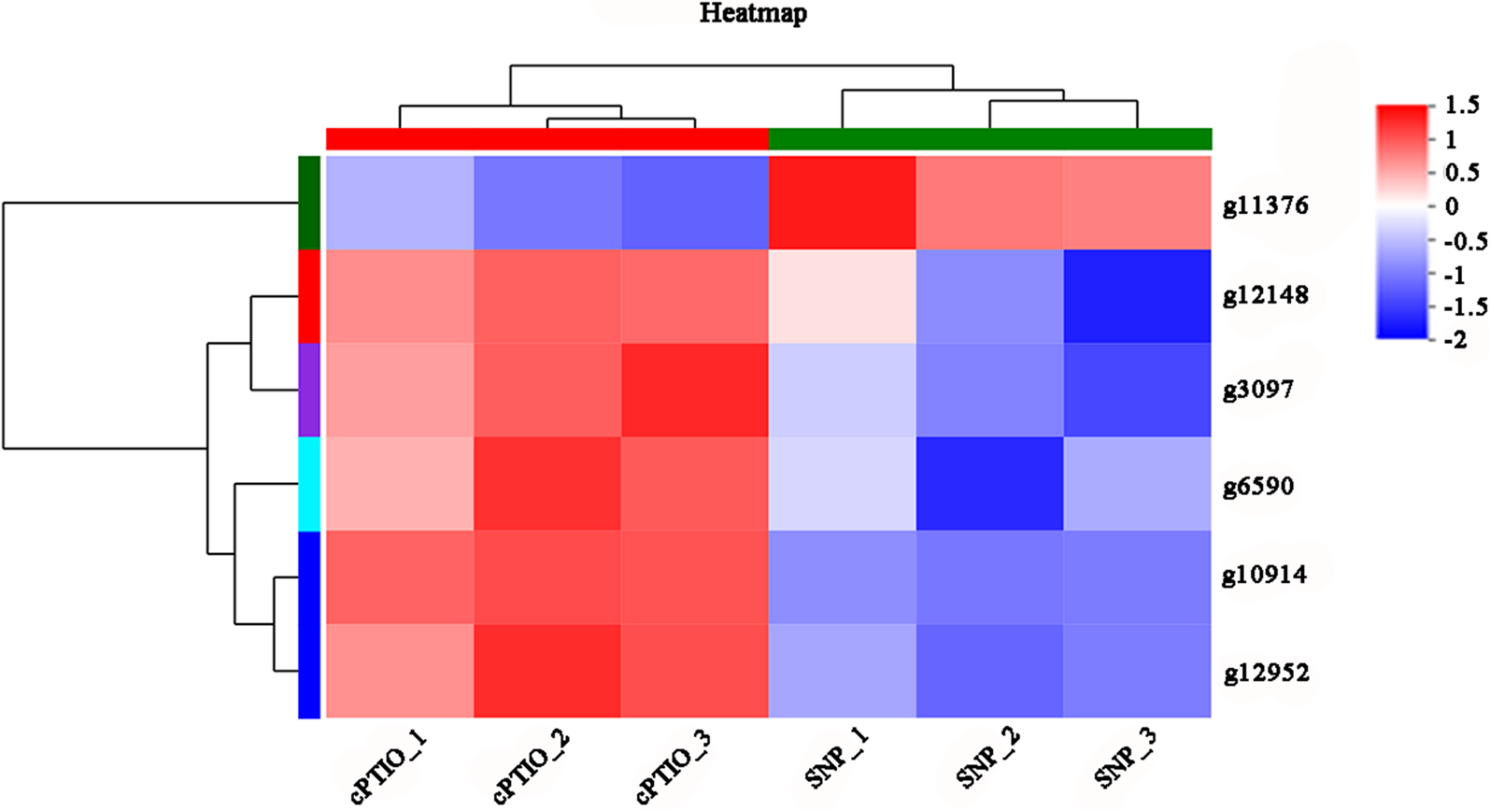
Heatmap of NO-regulated genes in the respiratory chain under HS

The respiratory chain is closely related to ROS. To further explore whether NO can regulate the respiratory chain to alleviate mycelial damage under HS, six DEGs related to the respiratory chain were identified from 579 DEGs via functional enrichment (Table 1). Two of these DEGs were not annotated, and the remaining four DEGs were g3097, g11376, g12148 and g12952, which encode the succinate dehydrogenase iron-sulfur subunit, AOX, hypothetical protein and the mitochondrial chaperone BCS1, respectively. As illustrated in the heatmap shown in Fig. 5, an exogenous NO donor (SNP) inhibited the expression of five of the DEGs and upregulated the expression of g11376 (AOX). The mitochondrial chaperone BCS1 is a transmembrane chaperone found in the mitochondrial inner membrane and it is required for the assembly of mitochondrial respiratory chain complex III (http://www.ebi.ac.uk/interpro/entry/InterPro/IPR027243/) [38]. The succinate dehydrogenase iron-sulfur subunit is involved in the synthesis and assembly of mitochondrial respiratory chain complex II (https://www.uniprot.org/uniprot/A1AZJ0). These results indicate that exogenous NO can inhibit the cytochrome pathway and activate the alternative oxidation pathway.

**Table 1:** List of genes related to the respiration that are regulated by NO

Gene ID	Gene description
g3097	Succinate dehydrogenase iron-sulfur subunit
g6590	–
g11376	Alternative oxidase
g12148	Hypothetical protein PLEOSDRAFT_1096427 (*P. ostreatus* PC15)
g12952	Mitochondrial chaperone BCS1
g10914	–

### NO induced ***aox*** gene expression under HS

ROS are mainly produced by the respiratory chain under high oxygen conditions and in a high reduction state during the transition of mitochondria from complex III to complex IV, which results in the leakage of a large number of electrons and the reduction of oxygen molecules. AOX prevents excessive reduction of downstream complexes (cytochrome pathway) by introducing a branch into the ETC at the ubiquinone pool. When AOX bypasses complexes III and IV of the cytochrome pathway, it significantly reduces ATP production and single electron leakage, which results in the reduction of ROS production [29]. Moreover, as determined through RNA-Seq analysis, *aox* can be regulated by NO, participates in the oxidation-reduction process pathway and has oxidoreductase activity.

**Fig. 6:**
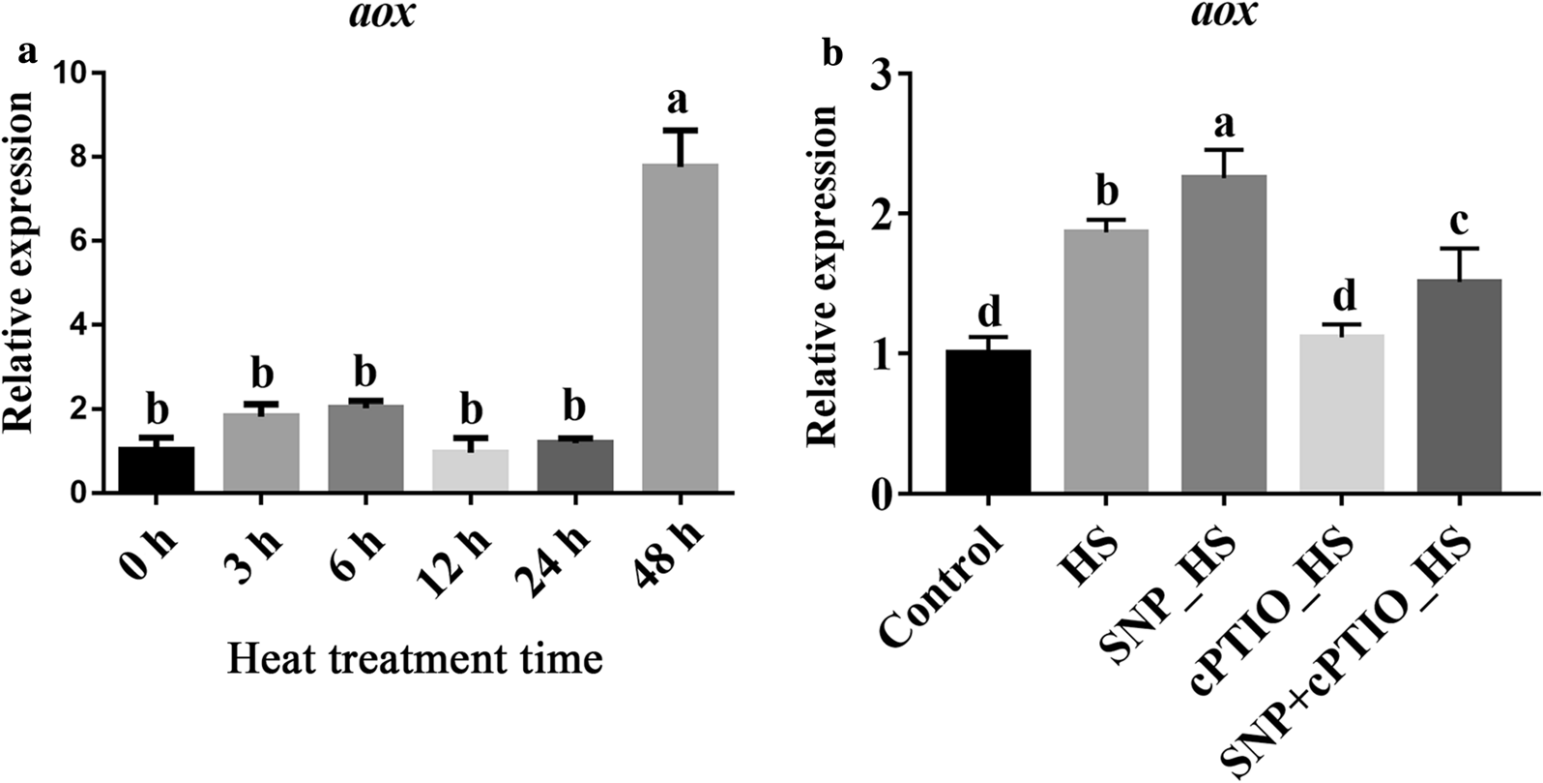
NO induced *aox* gene expression in *P. ostreatus* under HS. **A** Expression of the *aox* gene after exposure to HS for different amounts of time. **B** Effect of NO on the expression of the *aox* gene under HS. The values are presented as the means ± SEs from three independent experiments. Different letters indicate significant differences among the samples (*P* < 0.05 according to Duncan’s test)

To further verify the regulatory effect of NO on *aox* under HS, the effects of exogenous NO donors and scavengers on *aox* gene expression were assessed. As shown in Fig. 6A, the relative expression of the *aox* gene in *P. ostreatus* mycelia changed steadily with increases in the time of exposure to HS. During the first 24 h of exposure to HS, *aox* gene expression first increased and then decreased within a small range. An increase in the exposure time to 48 h significantly increased the relative expression of the *aox* gene, and the level detected after 48 h was approximately 8-fold higher than that at 0 h. As shown in Fig. 6B, the relative expression of the *aox* gene was significantly increased after HS (HS vs. control, *P* = 6.195 × 10^− 5^), whereas exogenous SNP treatment almost completely enhanced this effect (SNP vs. HS, *P* = 0.015), and cPTIO blocked the effect of SNP on *aox* gene expression (cPTIO vs. SNP, *P* = 5.704 × 10^− 6^). The results showed that NO can promote the expression of the *aox* gene in mycelia after HS. In conclusion, NO might participate in the response of *P. ostreatus* to HS by regulating the expression of the *aox* gene.

### The OE of ***aox*** promoted the recovery of mycelial growth after HS

**Fig. 7:**
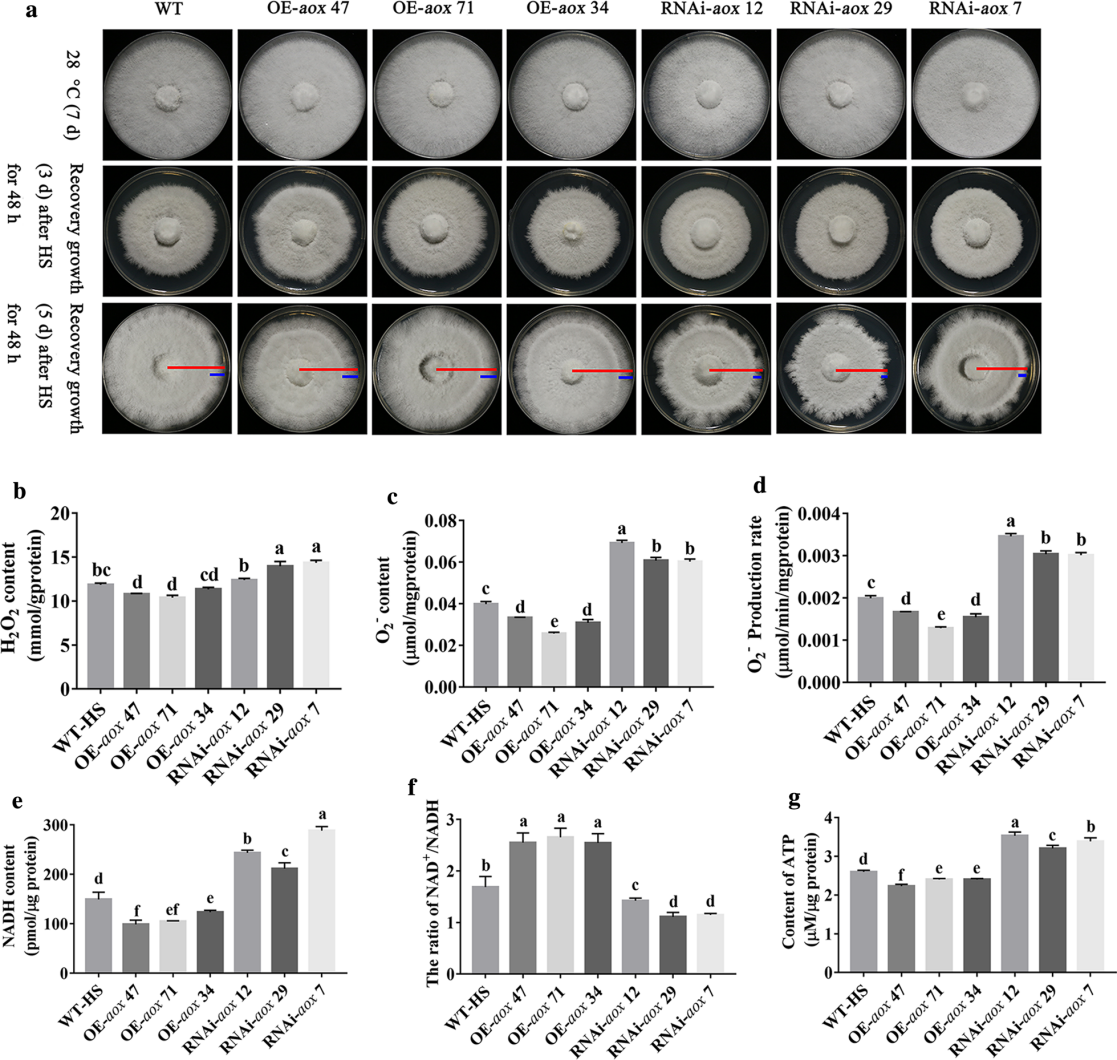
*aox* affects ROS production and promotes mycelial growth by regulating the respiratory pathway under HS. **A** Recovery of WT, OE-*aox* and RNAi-*aox* strains after HS. The red lines represent the radius of mycelial growth throughout the growth period, and blue lines represent the radius of mycelial growth during the 48 h of recovery after HS. **B** H_2_O_2_ content. **C** O_2_^−^ content. **D** O_2_^−^ production rate. **E** NADH content. **F** NAD^+^/NADH ratio. **G** ATP content. The values are presented as the means ± SEs from three independent experiments. Different letters indicate significant differences among the samples (*P* < 0.05 according to Duncan’s test)

Using previously reported methods [39], we successfully constructed an RNAi-*aox* plasmid (Additional file 1: Fig. S1) and established *aox*-transformed strains via *Agrobacterium-*mediated transformation. The *hyg* gene fragment was amplified for preliminary selection, and the relative expression of this target gene was amplified by quantitative reverse transcription PCR (RT-qPCR) to screen the RNAi-*aox* strains. The results are shown in Additional file 1: Fig. S2. To further explore the function of the *aox* gene in the response of *P. ostreatus* to HS, plates containing the various strains were cultured at 28 °C for 5 d, transferred to 40 °C for 48 h, and then incubated at 28 °C to allow growth recovery. After 3 d of growth recovery after HS, mycelial germination was observed in the wild type (WT), OE-*aox* and RNAi-*aox* strains, as shown in Fig. 7A. After 5 d of growth recovery, compared with the WT strain, the OE-*aox* strains exhibited a faster recovery rate and a complete colony edge, whereas RNAi-*aox* strains presented a slower mycelial recovery rate and showed defects on the edge of the colony. In conclusion, the *aox* gene plays an active role in the recovery of *P. ostreatus* mycelia after HS. Figure 7B, C and D show the changes in the H_2_O_2_ content, the O_2_^−^ content and the production rate of the *aox*-transformed strains under HS. Under HS, the accumulation of H_2_O_2_ in the OE-*aox* 47 and OE-*aox* 71 strains was significantly lower (by 9.05 and 12.28 %, respectively) than that in the WT strain. In addition, the H_2_O_2_ content in the OE-*aox* 34 strain was 4.29 % lower than that in the WT strain. In contrast, the H_2_O_2_ contents in the RNAi-*aox* 12, RNAi-*aox* 29 and RNAi-*aox* 7 strains were 4.59 %, 17.71 and 21.11 % higher, respectively, than that in the WT strain. As shown in Figs. 7C and D, the O_2_^−^ production rate and content of the OE-*aox* strains under HS were significantly lower than those of the WT strain, whereas those of the RNAi -*aox* strains increased significantly. These results indicated that the *aox* gene can regulate the production and accumulation of ROS. ROS are mainly caused by electron leakage in the respiratory chain. As shown in Figs. 7E and G, compared with the WT strain, the average NADH and ATP contents in the OE-*aox* strains were decreased by 26.47 and 9.82 %, respectively, and the average NAD^+^/NADH ratio in these strains was 1.55-fold higher. In contrast, the average NADH and ATP contents of the RNAi-*aox* strains were 66.86 and 29.79 % higher, respectively, than those in the WT strain, and the NAD^+^/NADH ratio was decreased by 26.52 %. Therefore, it can be speculated that the *aox* gene plays an important role in regulating the mitochondrial ETC and energy metabolism and in maintaining mitochondrial homeostasis.

In conclusion, the *aox* gene can affect the production and accumulation of ROS by regulating mitochondrial respiration, and it participates in the mycelial response to HS.

### The *aox* gene regulates the expression of key antioxidant enzyme genes in mycelia after HS

**Fig. 8:**
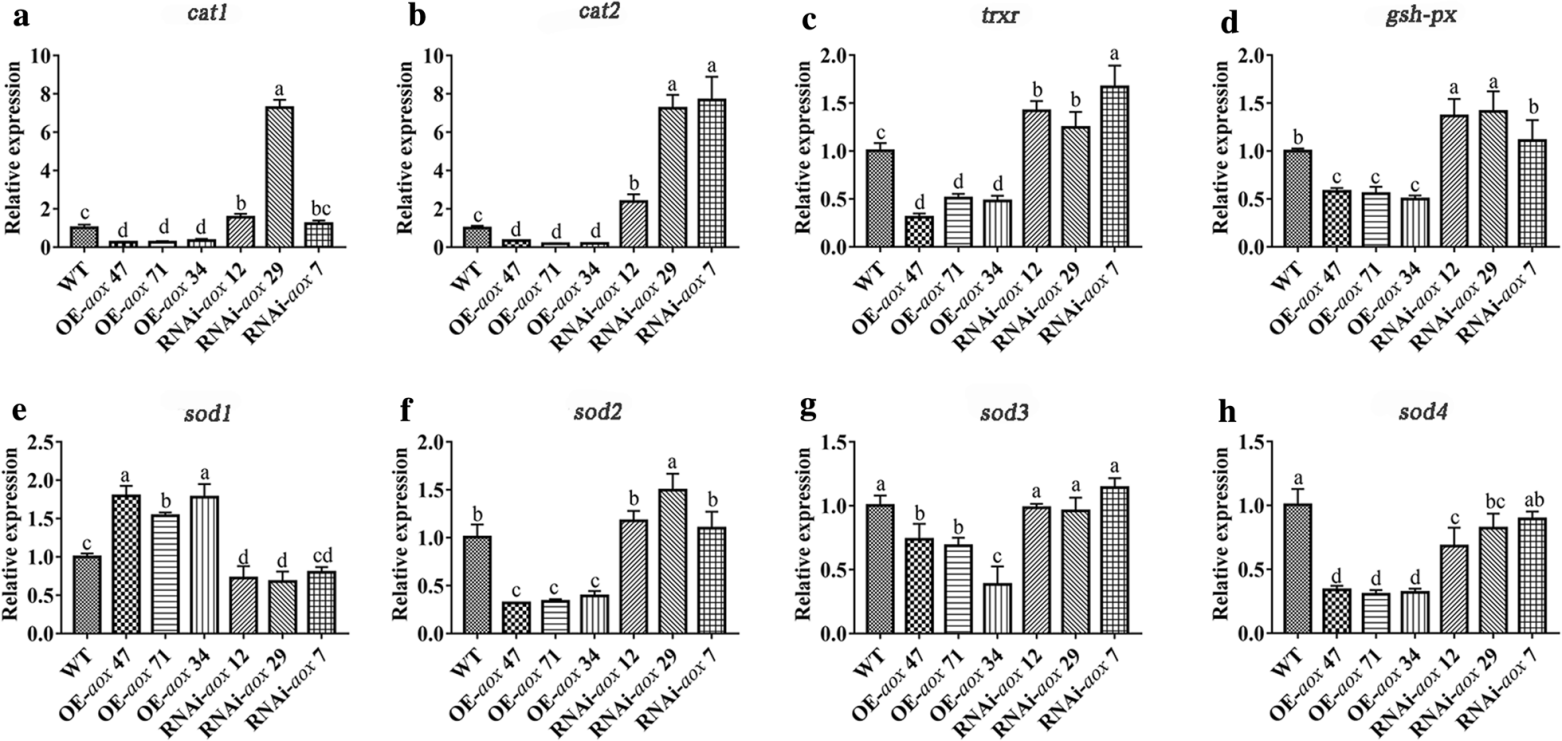
Expression of key antioxidant enzyme-encoding genes in *aox*-transformed strains under HS. **A** Relative expression of *cat1*. **B** Relative expression of *cat2.* **C** Relative expression of *trxr.* **D** Relative expression of *gsh-px.* **E** Relative expression of *sod1.* **F** Relative expression of *sod2.* **G** Relative expression of *sod3.* **H** Relative expression of *sod4.* The values are presented as the means ± SEs from three independent experiments. Different letters indicate significant differences among the samples (*P* < 0.05 according to Duncan’s test)

The signaling from organelles that controls nuclear gene expression is called retrograde signaling, and previous studies have shown that *aox* serves as a marker gene for mitochondrial retrograde regulation [40]. AOX also acts as a facilitator for signaling molecules conveying the metabolic status of mitochondria to the nucleus and is thus able to influence nuclear gene expression [41]. Moreover, studies have shown that AOX can affect the production and accumulation of ROS. Antioxidant systems (such as antioxidant enzymes and nonenzymatic oxidants) play critical roles in the defense against oxidative stress [42]. We measured the changes in expression of genes that encode four key antioxidant enzymes, namely, catalase (CAT), superoxide dismutase (SOD), thioredoxin reductase (TrxR) and glutathione peroxidase (GSH-PX). In the genome of *P. ostreatus*, two genes (*cat1* and *cat2*) encode CAT, four genes (*sod1*, *sod2*, *sod3* and *sod4*) encode SOD, one gene encodes TrxR, and one gene encodes GSH-PX [43]. To study whether *aox* in *P. ostreatus* can alleviate ROS stress by regulating the antioxidant enzyme system, we measured the expression of these key antioxidant enzyme genes in *aox*-transformed strains exposed to HS at 40 °C for 48 h. As shown in Fig. 8A, after 48 h of exposure to HS, the relative expression of *cat1* in the OE-*aox* 47-, OE-*aox* 71- and OE-*aox* 34-transformed strains was significantly downregulated to 26.5 %, 25.96 and 35.30 % respectively, of the level found in the WT strain. In the RNAi-*aox* 12 and RNAi-*aox* 29 strains, the relative expression of *cat1* was significantly increased by 1.57-fold and 7.28-fold, respectively, compared with that found in the WT strain, but no significant change in the expression of this gene was detected in the RNAi-*aox* 7 strain. As shown in Fig. 8B, compared with that in the WT strain, the relative expression of *cat2* in the OE-*aox* and RNAi-*aox* strains was significantly downregulated and upregulated, respectively. In conclusion, *aox* can negatively regulate *cat* gene expression under HS. As shown in Figs. 8C and D, the expression of the *trxr* and *gsh-px* genes was significantly downregulated in the OE-*aox*-transformed strains. In addition, the *trxr* gene was significantly upregulated in all RNAi-*aox* strains, and the relative expression of the *gsh-px* gene was significantly upregulated in RNAi-*aox*12 and RNAi-*aox* 29 and slightly upregulated in RNAi-*aox* 7. SOD is one of the major defense systems used to remove O_2_^−^. The relative expression levels of the four SOD-encoding genes in the *aox*-transformed strains under HS are shown in Figs. 8E–H. As shown in Fig. 8E, compared with the level in the WT strain, *sod1* gene expression was significantly increased in the OE-*aox* strains and decreased to 72.66 %, 67.71 and 79.93 % in RNAi-*aox* 12, RNAi-*aox* 29 and RNAi-*aox* 7, respectively. In addition, as shown in Figs. 8F–H, the expression levels of *sod2*, *sod3* and *sod4* in the OE-*aox* strains were significantly downregulated, to 34.93 %, 60.33 and 32.16 %, respectively, of the level found in the WT strain. However, *sod2* and *sod3* gene expression was not significantly changed in the RNAi-*aox* strains, and the expression of *sod4* was slightly downregulated in these strains compared with the WT strain. Because the *sod1* gene encodes Gu-SOD, which mainly exists in the cytoplasm, it can be concluded that the OE of *aox* during HS can regulate the expression of SOD-encoding genes.

In conclusion, in addition to SOD1, the OE-*aox* strains exhibited downregulated expression of key antioxidant enzyme coding genes under HS (40 °C for 48 h), and the RNAi-*aox* strains exhibited upregulated expression of CAT-, GSH-PX- and TrxR-encoding genes. It can thus be hypothesized that the significant upregulation of the *aox* gene after HS (40 °C for 48 h) can further affect the expression of key antioxidant enzyme-encoding genes.

### Exogenous benzohydroxamate (BHAM) regulated the expression of antioxidant enzyme-encoding genes in mycelia under HS

AOX is insensitive to inhibitors of the cytochrome pathway such as cyanide, antimycin A, and miyxothiazol, but is inhibited by primary hydroxamic acids, such as BHAM [44, 45]. To further prove whether *aox* interference can regulate the expression of key antioxidant enzyme-encoding genes, an experiment in which different concentrations of an AOX inhibitor (BHAM) were added was then performed. The results showed that the growth rate of *P. ostreatus* mycelia at 28 °C was affected by exogenous BHAM. As shown in 1: Figs. S3A, B, a low concentration of BHAM (50–100 µM) had no significant effect on the colony and mycelial growth rate of *P. ostreatus*, but the addition of exogenous BHAM at a concentration higher than 200 µM affected overall mycelial growth and significantly reduced the mycelial growth rate. Furthermore, whether exogenous BHAM could regulate the expression of the *aox* gene was assessed in the experiment. As shown in Additional file 1: Fig. S3C, the addition of BHAM at a concentration of 50–200 µM significantly downregulated the expression of the *aox* gene compared with the control (CK) level, which indicated that low concentrations of BHAM can inhibit the expression of the *aox* gene. However, a BHAM concentration of 400 µM upregulated the expression of the *aox* gene, and because this concentration also significantly inhibited the mycelial growth rate, it can be speculated that 400 µM BHAM might affect the growth environment of mycelia and induce abiotic stress, which eventually leads to the induction of *aox* gene expression. In addition, because the mycelial growth of the RNAi-*aox* strains did not significantly differ from the normal growth of *P. ostreatus* mycelia, 50–200 µM was used as the BHAM concentration in the subsequent experiments.

**Fig. 9:**
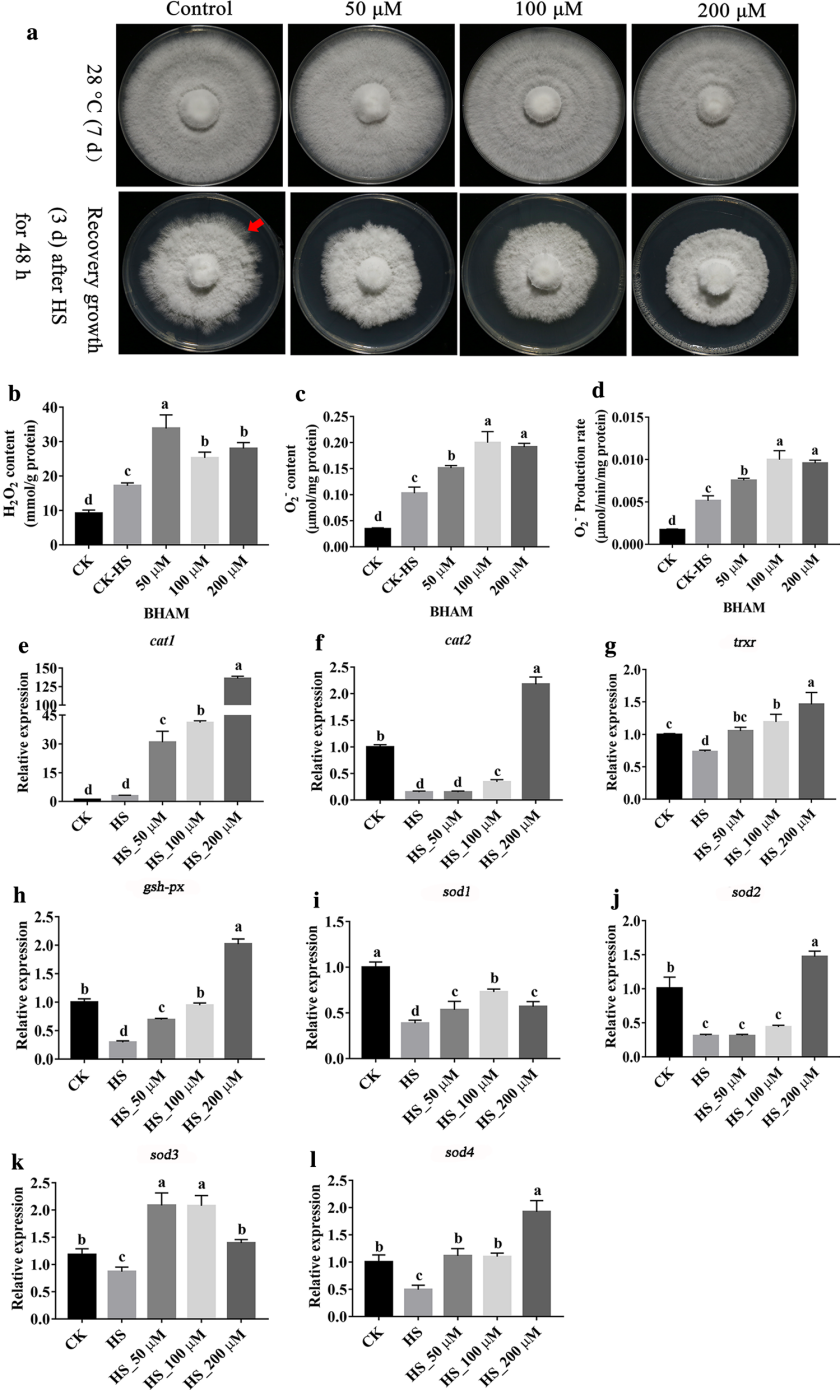
Exogenous BHAM regulates the expression of antioxidant enzymes under HS. **A** Exogenous BHAM inhibited the growth of mycelial growth after HS. The red arrow points to the mycelia that experienced growth recovery. **B** H_2_O_2_ content. **C** O_2_^−^ content. **D** O_2_^−^ production rate. **E** Relative expression of *cat1*. **F** Relative expression of *cat2.* **G **Relative expression of *trxr.* **H** Relative expression of *gsh-px.* **I** Relative expression of *sod1.* **J** Relative expression of *sod2.* **K** Relative expression of *sod3.* **L** Relative expression of *sod4.* The values are presented as the means ± SEs from three independent experiments. Different letters indicate significant differences among samples (*P* < 0.05 according to Duncan’s test)

As shown in Fig. 9A, the addition of exogenous BHAM at different concentrations slowed the recovery of mycelial growth after HS compared with that of the WT strain, and this result was consistent with the results obtained with the RNAi-*aox* strains. The effects of exogenous BHAM on the H_2_O_2_ content and the O_2_^−^ content and production rate were then assessed. The results showed that compared with the CK-HS group, the H_2_O_2_ content and O_2_^−^ content and production rate of mycelia under HS were significantly increased by different concentrations of BHAM (Figs. 9B and D). These results indicated that exogenous BHAM could promote the production and accumulation of ROS in mycelia under HS. The effects of exogenous BHAM on the expression of key antioxidant enzyme-encoding genes in mycelia under HS are shown in Figs. 9E–L. As illustrated in the figures, the addition of exogenous BHAM significantly increased the expression of antioxidant enzyme-encoding genes under HS. Specifically, the expression levels of *cat1* (Fig. 9E), *cat2* (Fig. 9F), *trxr* (Fig. 9G), *gsh-px* (Fig. 9H), *sod2* (Fig. 9J) and *sod4* (Fig. 9L) increased with increases in the BHAM concentration, and the highest expression levels of these genes, which were 47.25-fold, 17.76-fold, 1.99-fold, 6.86-fold, 4.76-fold and 3.98-fold higher than those found in the HS group, respectively, were detected with a BHAM concentration of 200 µM. In addition, the highest expression levels of *sod1* and *sod3*, which were 1.90-fold and 2.42-fold higher than the control levels, respectively, were obtained with the exogenous addition of 100 µM BHAM (Figs. 9I and K).

In conclusion, exogenous BHAM can promote the production and accumulation of ROS under HS and then regulate the expression of antioxidant enzyme-encoding genes.

## Discussion

NO is a type of free radical involved in many types of stress and physiological processes and serves as a key regulator of physiological processes [46]. In this study, we revealed that NO reduces the production of ATP and ROS by activating the alternative oxidation pathway, maintaining mitochondrial function, and enhancing the resistance of *P. ostreatus* to HS. In addition, AOX can act on the retrograde signaling pathway to regulate the expression of nuclear genes (antioxidant enzyme-encoding genes) in response to HS. This finding provides a new perspective on the fungal regulatory mechanism of NO in response to abiotic stress.

The regulatory effect of NO on metabolism during stress relief is not unusual. Recently, an increasing number of studies have shown that the application of exogenous NO is useful for allaying oxidative stresses caused by drought, high temperature and salinity [47–49]. HS refers to an increase in temperature to a level that exceeds the optimal growth temperature of the organism of interest, which causes irreversible damage to its growth [50]. HS induces an increased generation of ROS. The production rate of ROS is strongly dependent on the membrane potential, and a change in the membrane potential increases the probability of electron leakage and thus the production of O_2_^−^, which can be used as a substrate to produce H_2_O_2_ and hydroxyl radicals [51]. In addition, excessive production and accumulation of ROS can cause lipid peroxidation, membrane leakage, enzyme inactivation, and DNA fragmentation or mutation, which can result in serious cell damage [52, 53]. During its cultivation, *P. ostreatus* is often exposed to high temperature. The results obtained in this study show that exogenous NO can reduce the content of NADH and the production of ATP under HS. Previous studies have shown that NO can regulate the NAD^+^/NADH ratio in *Ganoderma lucidum* under HS [54], which is consistent with our results. Second, the NAD^+^/NADH ratio is mainly regulated by the TCA cycle, and NADH serves as a substrate for the generation of ATP in the respiratory chain [37]. Therefore, it can be speculated that exogenous NO can affect ATP production by regulating the NADH content. In addition, the production and accumulation of ROS in mycelia was decreased by exogenous NO addition. Previous studies have shown that NO can reduce ROS accumulation under HS by regulating different pathways. For instance, in wheat, the addition of exogenous NO donors can activate antioxidant enzymes, which results in the reduction of ROS and improvements in the heat resistance of wheat coleoptiles [55]. In rice, NO might protect photosynthesis from HS and alleviate oxidative stress by scavenging ROS [56]. In yeast, NO may be involved in various stress response systems, including H_2_O_2_-induced apoptosis [57]. For example, in *Saccharomyces cerevisiae*, the activation of Mac1 transcription factors by NO produced under a high temperature-stress conditions is important for the activation of the copper-dependent SOD1 [58]. The results of this study are consistent with those reported in previous studies. Because ROS are mainly caused by electron leakage in the respiratory chain, it can be speculated that exogenous NO can reduce the production and release of ROS under HS by regulating the respiratory chain.

Stress-induced gene expression changes are key components of the molecular mechanism underlying plant adaptation to environmental challenges [59]. In recent years, RNA-Seq has become an effective means to study changes in gene expression under stress. For example, in *Camellia sinensis*, a total of 89 putative AP2/ERF transcription factors related to the temperature response were identified by RNA-Seq [60]. Under drought stress, 5689 DEGs in *Populus trichocarpa* leaves were obtained by RNA-Seq analysis, including genes related to the drought stress response and involved in photosynthesis, cell wall organization, and osmoprotectant metabolism [61]. Therefore, RNA-Seq is an effective method for exploring the stress response mechanisms of organisms based on changes in gene expression. In this study, RNA-Seq was used to further explore the response mechanism through which NO alleviates mycelial damage under HS. A total of 579 DEGs specifically regulated by NO were identified. These genes might participate in the response of *P. ostreatus* mycelia to HS by regulating cell metabolism, affecting the composition and structure of the cell membrane, or modifying the catalytic activity of proteins. Interestingly, the functional enrichment analysis identified 6 DEGs among the 579 DEGs that were not only regulated by NO but also closely related to the respiratory chain. Among these 6 DEGs, mitochondrial chaperone BCS1 (g12952) and the succinate dehydrogenase iron-sulfur subunit (g3097) are necessary for complexes II and III, respectively, which are closely related to the cytochrome pathway, and AOX (g11376) is involved in the alternative oxidation pathway. Further analysis showed that only AOX was upregulated after SNP addition, whereas the other DEGs were downregulated. In *P. ostreatus*, NO can induce *aox* gene expression by inhibiting the expression of aconitase gene and protein, which results in citric acid accumulation [20]. This finding is consistent with our previous results. In plants, it has also been reported that NO can regulate the expression of the *aox* gene. For instance, Fu et al. showed that NO is an inducer of *aox* in tobacco plants infected with *tobacco mosaic virus* [62], and another study found that *aox* is induced in *Arabidopsis* cell suspensions treated with NO [63]. Therefore, NO can induce *aox* gene expression in both fungi and plants.

AOXs have been documented to occur in plants [64], algae [65], yeasts and pathogenic fungi, such as *A. fumigatus* [66], *Histoplasma capsulatum* [67], and *Paracoccidioides brasiliensis* [68]. AOX is a nonenergy-conserving terminal oxidase in the mitochondrial ETC, and previous studies have shown that AOX functions to balance energy stability and keep the ETC flowing through mitochondria by limiting the formation of mitochondrial ROS [35, 36]. In plants, *aox* plays important roles in countering abiotic stresses, including drought, light, temperature and salinity [32, 69, 70]. For example, in tobacco, under drought stress, the recoverability of *aox* knockdown plants is strongly compromised [71]. In fungi, it has been reported that *aox* is upregulated under mitochondrial respiratory chain inhibition or oxidative stress conditions [67, 72]. For example, in *P. brasiliensis*, *aox* expression is also upregulated by ROS generation and mitochondrial respiratory chain inhibitors, and heterologous expression of *aox* in *S. cerevisiae* decreases intracellular ROS generation [68]. In the presence of O_2_^−^, *aox* in *A. fumigatus* may play a role in antioxidant defense mechanisms [73]. In this study, the OE-*aox* strains rapidly resumed their growth after HS, and *aox* OE could reduce the contents of NADH and ATP and the content and production rate of O_2_^−^. In contrast, the opposite results were obtained with the RNAi-*aox* strains. Previous studies with *Arabidopsis thaliana* have shown that under hypoxic stress, *aox* can regulate energy metabolism, affect ROS production and enhance resistance [74], and we obtained similar results. The formation of ROS is mainly due to “single electron leakage” of respiratory chain components, and complexes I and III are considered the main sites of this electron leakage [75]. Therefore, it can be speculated that the *aox* gene might reduce the production of O_2_^−^ by regulating the respiratory pathway and maintaining mitochondrial homeostasis and function in *P. ostreatus*.

A large number of studies have shown that AOX mediates a retrograde signaling pathway that in turn regulates gene expression both transcriptionally and post transcriptionally in response to stress. AOX serves as a link between metabolic activities (mitochondria) and signaling (nucleus) [41, 76]. In this study, the expression of antioxidant enzyme-encoding genes in the OE-*aox* strains was downregulated after HS, whereas the expression of these genes in the RNAi-*aox* strains was upregulated, and these findings might be related to the regulation of ROS production by *aox*. BHAM is an inhibitor of AOX, and exogenous BHAM also promoted the expression of antioxidant enzyme-encoding genes under HS, which was very similar to the results obtained with the RNAi-*aox* strains. Therefore, it can be speculated that in response to HS, the *aox* gene can mediate the retrograde signaling pathway in *P. ostreatus* mycelia to regulate the expression of antioxidant enzyme-encoding genes.

## Conclusions

**Fig. 10:**
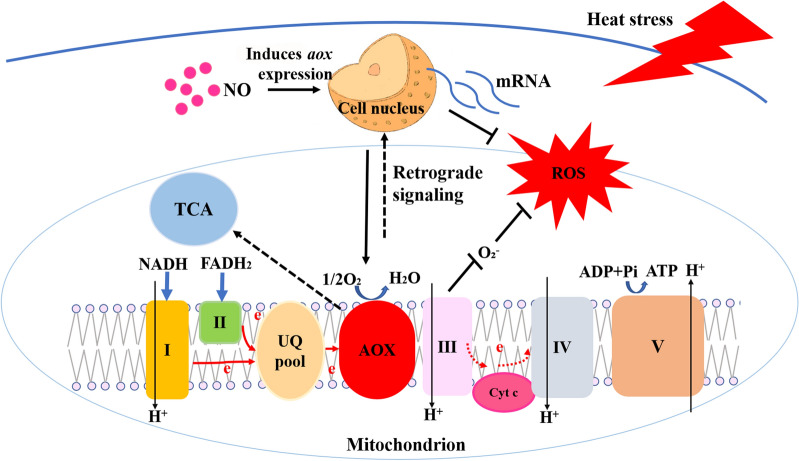
Schematic representation of the mechanism through which NO alleviates ROS production by inducing *aox* gene expression under HS. I, II, III, IV, and V: Complexes 1–5, respectively

In summary, our data indicate that NO responds to HS by reducing the production and accumulation of ROS in *P. ostreatus* mycelia and can induce *aox* gene expression. The construction of OE-*aox* and RNAi-*aox* strains showed that high *aox* gene expression can affect cell respiration by reducing the content of NADH, which is a product of the TCA cycle, thereby reducing the production and accumulation of ROS. Further studies showed that under HS, *aox* can mediate the reverse signaling pathway to regulate the expression of antioxidant enzyme genes in *P. ostreatus*, thereby regulating the mycelial response to ROS. Based on these findings, we proposed a potential cascade of cellular events comprising the NO-mediated alleviation of ROS production via *aox* gene expression (Fig. 10). Our study improves the understanding of the biological functions and regulatory pathways of NO in *P. ostreatus* under HS and provides new ideas for further studies on the functions of AOX in fungi.

## Methods

### Strains and growth conditions

The *P. ostreatus* CCMSSC 00389 strain was provided by the China Center for Mushroom Spawn Standards and Control. The WT, OE-*aox*, and RNAi-*aox* strains were incubated on PDA plates. *Agrobacterium tumefaciens* GV3101 (IMCAS, Beijing, China) was grown in Luria-Bertani (LB) medium (Oxoid, England) containing 100 µg/mL kanamycin (VWR Life Science, USA) and 50 µg/mL rifampin (MP Biomedicals, France) and was used to transform *P. ostreatus*. *Trans* 1-T1 phage-resistant chemically competent cells (TransGen Biotech, Beijing, China) were used for plasmid construction and were grown in LB broth containing kanamycin (50 µg/mL).

### HS and growth recovery of mycelia

The WT, OE-*aox* 47, OE-*aox* 71, OE-*aox* 34, RNAi-*aox* 12, RNAi-*aox* 29 and RNAi-*aox* 7 strains were used in this study. According to the research methods used in our previous study [4], the different strains were incubated at 28 °C for 5 d, treated at 40 °C, and then returned to growth at 28 °C. Some of the plates were used to collect mycelia, and the collected mycelia were rapidly placed in liquid nitrogen and stored in a freezer at − 80 °C for subsequent experiments. After HS, the rest of the strains were placed at 28 °C to allow them to resume growth, and mycelial growth after 3 and 5 d was observed and recorded.

For the exogenous BHAM addition experiment described in this paper, the WT strain was cultured at 28 ℃ for 5 d on PDA plates with different concentrations of BHAM (control, 50 µM, 100 µM, 200 µM). Then, the PDA plates of different treatment groups were transferred to 40 ℃ for 48 h and finally placed at 28 ℃ to resume growth for 3 d.

### Determination of the protein and H_2_O_2_ content

Protein concentrations were determined using the Bradford Protein Quantification Kit (Vazyme, Nanjing, China), with bovine serum albumin as the standard, following the manufacturer’s instructions. The intracellular H_2_O_2_ content was determined using an H_2_O_2_ Quantitative Assay Kit (Sangon Biotech, Shanghai, China) according to the manufacturer’s instructions. In an acidic environment, H_2_O_2_ can oxidize Fe^2+^ to Fe^3+^, and then Fe^3+^ combines with dye molecules to form a complex, which has the maximum absorption wavelength at 595 nm. The absorbance at 405 nm was detected by a microplate reader (Tecan Infinite®M200 Pro, Switzerland) to determine the amount of complex formation, and then the content of H_2_O_2_ was calculated.

### Determination of the O_2_^−^ content and production rate

Excessive accumulation of O_2_^−^ will destroy the cell membrane. The content and production rate of O_2_^−^ in the mycelia of the different strains subjected to the different treatments were detected using an O_2_^−^ Content Detection Kit (Solarbio, Beijing, China) according to the manufacturer’s instructions. O_2_^−^ reacts with hydroxylamine hydrochloride to form nitrite ions, which form a red compound under the action of p-aminobenzenesulfonic acid and naphthalene ethylenediamine hydrochloride, and this compound has a characteristic absorption peak at 530 nm. The content and production rate of O_2_^−^ in mycelia were then calculated based on the absorbance value at 530 nm obtained using a microplate reader (Tecan Infinite®M200 Pro, Switzerland).

### Determination of the NADH and NAD^+^ content

Nicotinamide adenine dinucleotide (NAD) is a coenzyme that exists in all cells and is found in two forms: oxidized (NAD^+^) and reduced (NADH). In this study, the changes in the NADH content and the NAD^+^/NADH ratio in the WT and *aox*-transformed strains under HS were determined using an NAD^+^/NADH Assay Kit (Beyotime, Shanghai, China) according to the manufacturer’s instructions. Ethanol is oxidized to acetaldehyde in the presence of alcohol dehydrogenase, and during this process, NAD^+^ is reduced to NADH. Subsequently, NADH reduces 2-(2-methoxy-4-nitrophenyl)-3-(4-nitrophenyl)-5-(2,4-disulfobenzene)-2 H-tetrazole to orange formazan in the presence of 1-methoxy-5-methylpenazinium methyl sulfate, and the product has a characteristic absorption peak at 450 nm. The samples from the different treatment groups were ground up with liquid nitrogen, and 30 mg was added to 400 µL of extraction solution, homogenized, incubated on ice for 10 min, and centrifuged at 12,000 *g* and 4 °C for 10 min. The supernatant was then collected. The total amounts of NAD^+^ and NADH were determined according to the instructions provided with the kit, and the NAD^+^/NADH ratio was calculated.

### Determination of the ATP content and total respiratory rate

Respiration is the core process of mitochondrial metabolism, and a large amount of free energy is released by oxidative phosphorylation for the production of ATP [77]. In this study, the total respiratory rate was measured according to previous studies [78]. Specifically, the changes in the ATP content during different treatments were measured using an enhanced ATP Assay Kit (Beyotime, Shanghai, China) according to the manufacturer’s instructions. This assay is based on the fact that firefly luciferase needs ATP to provide energy for the production of fluorescence. The samples were lysed with the ATP extract in the kit, homogenized for 10 min and centrifuged at 12,000 *g* and 4 °C for 10 min. The supernatant was then used to determine the ATP content and protein concentration, and the ATP content in different samples was detected by multifunction microplate reader (Tecan Infinite®M200 Pro, Switzerland).

### RNA extraction, cDNA library construction, and RNA sequencing (RNA-Seq)

SNP is a donor of NO, and cPTIO is a scavenger of NO. To understand the regulatory mechanism through which NO alleviates the mycelial damage in *P. ostreatus* induced by HS, four different treatment groups (CK, HS, SNP_HS and cPTIO_HS) were established for RNA-Seq analysis, with 3 replicates in each group and 12 samples in total. For the preparation of RNA-Seq samples described in this paper, the WT strains were cultured at 28 °C on PDA plates for 5 d as the CK group and then transferred to new PDA plates with 100 µM SNP (SNP_HS) or 250 µM cPTIO (cPTIO_HS) treatment according to a previous study [20], after which they were treated with HS(40 °C) for 48 h. Meanwhile, some WT strains cultured at 28 ℃ for 5 d were transferred to the new PDA plates for HS treatment (40 °C, 48 h) as the HS group.

Total RNA was extracted from the mycelia of *P. ostreatus* using the TRIzol® Reagent (Invitrogen, USA), according to the manufacturer’s instructions, and genomic DNA was removed using DNase I (TaKaRa, Japan). The RNA quality was determined using a 2100 Bioanalyzer (Agilent, CA, USA). The RNA-Seq transcriptome library was prepared using the TruSeq™ RNA sample preparation kit from Illumina (San Diego, CA,USA) and 1 µg of total RNA. Briefly, messenger RNA was isolated according to the polyA selection method using oligo (dT) beads and then fragmented using fragmentation buffer. Double-stranded cDNA was then synthesized using a SuperScript double-stranded cDNA synthesis kit (Invitrogen, CA, USA) with random hexamer primers (Illumina). The synthesized cDNA was then subjected to end repair, phosphorylation and ‘A’ base addition according to the library construction protocol established by Illumina. The libraries were subjected to size selection to obtain cDNA target fragments of 200–300 bp using 2 % Low Range Ultra Agarose followed by 15 cycles of PCR amplification with Phusion DNA polymerase (NEB). After quantification by TBS380, the paired-end RNA-Seq library was sequenced with an Illumina NovaSeq 6000 sequencer (2 × 150-bp read length), and the sequencing data were deposited into the Sequence Read Archive of the National Centre for Biotechnology Information (NCBI) with the accession number SRP277542.

### Analysis of DEGs

To identify the DEGs between the CK and HS, SNP_HS and cPTIO_HS groups, the expression level of each transcript was calculated using the fragments per kilobase of exon per million mapped reads (FRKM) method. RSEM (http://deweylab.github.io/RSEM/) was used to quantify the gene abundances [79]. Differential expression analysis was performed using edgeR software in the R statistical package [80]. In addition, the GO functional enrichment of the DEGs was analyzed.

### Construction of RNAi- *aox* plasmid and strains

Previous studies have shown that gene knockout and gene transformation using vectors constitute an effective strategy for exploring the function of fungal genes. In addition, OE-*aox* strains were successfully obtained in our previous study [20]. In this study, the original pCAMBIA1300 vector was modified to harbor the hyg phosphotransferase gene (*hyp*), which was expressed under the control of the upstream lac promoter. The RNAi-*aox* plasmid was constructed as follows, the *P. ostreatus* gpd promoter was PCR amplified, after which the *aox*-sense and *aox*-antisence were amplified using primers with homologous arms, as shown in Additional file 1: Table S1. Homologous recombination was then performed to connect the target fragments to the plasmid one by one. The constructed plasmid map is shown in Additional file 1: Fig. S1. The *aox*-JC-F/R primers (Additional file 1: Table S1) were used to amplify the target fragment and thus detect whether the interference plasmid was successfully constructed. Finally, the RNAi-*aox* plasmid was transformed into *P. ostreatus* mycelia by *A. tumefaciens* GV3101 according to our previous transformation method [39]. The *hyg* fragment was amplified to screen the transformed strains, and RT-qPCR was performed to identify the strains with high interference for further experiments.

### RNA extraction, reverse transcription and RT-qPCR

Total RNA was extracted using an E.Z.N.A. Plant RNA Kit (Omega Bio-tek, Norcross, GA, USA) following an extraction method for fungal samples. First-strand cDNA was synthesized using the HiScript II 1st Strand cDNA Synthesis Kit (Vazyme, Nanjing, China) according to the manufacturer’s instructions. The ChamQ SYBR RT-qPCR Master Mix Kit (Vazyme, Nanjing, China) and the ABI 7500 real-time PCR amplifier (Applied Biosystems, Foster City, CA, USA) were used for RT-qPCR. According to our previous study [81], the RT-qPCR amplification procedure was as follows: 95 °C for 3 min, 40 cycles at 95 °C for 3 s and at 60 °C for 32 s, and a final extension at 72 °C for 30 s. In this study, RT-qPCR was performed to analyze the mRNA expression levels of the *aox* gene and key antioxidant enzyme genes in the CCMSSC00389 strain and *aox*-transformed strains subjected to the different treatments. The *β-actin* and *β-tubulin* genes were used as internal reference genes, and the relative gene expression was determined according to the 2^−△△CT^ method.

### Statistical analysis

In this study, at least three biological replications were performed in each group. All data were recorded using Excel 2010 software (Microsoft, Redmond, WA, USA). Duncan’s multiple range test was used for all significance analysis, and a *P* value < 0.05 was considered significant by SPSS software (SPSS Inc., Chicago, IL, USA) [82]. All figures were generated using GraphPad Prism 6 (GraphPad Software Company, San Diego, California, USA).

## Supplementary Information

**Additional file 1: Fig. S1.** Structure of the RNAi-*aox* vector. **Fig. S2** Validation and selection of RNAi-*aox* strains. **A** Preliminary selection of RNAi-*aox* transformed strains by amplification of the *hyg* gene. **B** RT-qPCR analysis of the expression of *aox* in the tested strains. **Fig. S3** Regulation of mycelial growth and *aox* gene expression by different concentrations of BHAM. **A** Effects of different concentrations of BHAM on colony morphology.** B** Effects of different concentrations of BHAM on the mycelial growth rate **C** Effects of different concentrations of BHAM on *aox* gene expression in mycelia. The values are presented as the means ± SEs from three independent experiments. Different letters indicate significant differences among the samples (*P* < 0.05 according to Duncan’s test).**Table S1** Primers used in this study. **Table S2** Reads mapped to the reference *P. ostreatus* CCMSSC 00389 genome.

## Data Availability

The datasets used and analysed during the current study available from the corresponding author on reasonable request.

## Abbreviations

*P. ostreatus*: *Pleurotus ostreatus*
NO: Nitric oxide
*aox*: Alternative oxidase
HS: Heat stress
TCA: Tricarboxylic acid
ROS: Reactive oxygen species
Ca^2+^: Calcium
H_2_O_2_: Hydrogen peroxide
ETC: Electron transport chain
OE: Overexpression
RNAi: RNA interference
O_2_^−^: Superoxide anion
NAD: Nicotinamide adenine dinucleotide
ATP: Adenosine triphosphate
NCBI: National Centre for Biotechnology Information
SNP: Sodium nitroprusside
cPTIO: 2-(4-carboxyphenyl)-4,4,5,5-tetramethylimidazoline-1-oxyl3-oxidec
FRKM: Fragments per kilobase of exon per million mapped reads
DGEs: Differentially expressed genes
GO: Gene ontology
RT-qPCR: Quantitative reverse transcription PCR
RNA-Seq: RNA sequencing
CAT: Catalase
SOD: Superoxide dismutase
TrxR: Thioredoxin reductase
GSH-PX: Glutathione peroxidase
BHAM: Benzohydroxamate
