# Supplementary material for: Alternative oxidase gene induced by nitric oxide is involved in the regulation of ROS and enhances the resistance of Pleurotus ostreatus to heat stress
Source: Microb Cell Fact. 2021 Jul 19;20:137. doi: 10.1186/s12934-021-01626-y (PMC8287771; doi:10.1186/s12934-021-01626-y)
Supplement: Supplementary file 1 — Additional file 1: Fig. S1. Structure of the RNAi-aox vector. Fig. S2 Validation and selection of RNAi-aox strains. A Preliminary selection of RNAi-aox transformed strains by amplification of the hyg gene. B RT-qPCR analysis of the expression of aox in the tested strains. Fig. S3 Regulation of mycelial growth and aox gene expression by different concentrations of BHAM. A Effects of different concentrations of BHAM on colony morphology. B Effects of different concentrations of BHAM on the mycelial growth rate C Effects of different concentrations of BHAM on aox gene expression in mycelia. The values are presented as the means ± SEs from three independent experiments. Different letters indicate significant differences among the samples (P < 0.05 according to Duncan’s test).Table S1 Primers used in this study. Table S2 Reads mapped to the reference P. ostreatus CCMSSC 00389 genome. [file 12934_2021_1626_MOESM1_ESM.docx]

**Additional file 1**

**Table S1** Primers used in this study.

| Primer | Sequence (5′→3′) | Note |
| --- | --- | --- |
| *cat1*-qF | TGTGCATTGGTTGAGAGAGG | qPCR |
| *cat1*-qR | TACGACGCTACAACTTCCG |  |
| *cat2*-qF | CGGACTTTCTTGCCCACAG |  |
| *cat2*-qR | GACTTGCTCGCCCATTTCG |  |
| *SOD1*-qF | CCTCATCTTCAACTACGCAAGT |  |
| *SOD1*-qR | GCAGACGAACCACAGGAATC |  |
| *SOD2*-qF | ACACGAAGCATCATCAGACCTA |  |
| *SOD2*-qR | GAAGAGCGAGTGGTTGATATGG |  |
| *SOD3*-qF | GTTCTCCTAGCAGCGAAGA |  |
| *SOD3*-qR | CATTCCCCGTTTTAAGTGAC |  |
| *SOD4*-qF | TTGAACGAGACTTTGGCACC |  |
| *SOD4*-qR | ATGATCGGCGCGTGAGTTATC |  |
| *GSH-PX*-qF | AAGGTCTTCAGGCATTGTAT |  |
| *GSH-PX*-qR | GTGGTTACGCTCACAGAA |  |
| *TrxR*-qF | GACGGCTACATCATCACC |  |
| *TrxR*-qR | AATGAGCTTCTCGACCTC |  |
| *aox*-qF | CTCCACAGATCCACACTCTTC |  |
| *aox*-qR | CCGCCTCAAAGTCATAAAAGTC |  |
| *β-actin*-F | AGTCGGTGCCTTGGTTAT | Endogenous control |
| *β-actin*-R | ATACCGACCATCACACCT |  |
| *β-tublin*-F | AGGCTTTCTTGCATTGGTACACGC |  |
| *β-tublin*-R | TATTCGCCTTCTTCCTCATCGGCA |  |
| *aox*-RNAi-F1 | CCATCTCCTCAGATCTTGTTGGGAAGGGCCAGGC | Construction of RNAi plasmids |
| *aox*-RNAi-R1 | TAAGCTCTAAACTAGTCTGAAAAGGGATGAAGGGCGG |  |
| *aox*-RNAi-F2 | CCCTTTTCAGACTAGTGTTAAGCCATGCATTCTGATCCAGG |  |
| *aox*-RNAi-R2 | CAATTCTAGAGGGCCCTGTTGGGAAGGGCCAGGC |  |
| *aox-*JC-F | CCGACATATCTGCTCAT | Detection of RNAi plasmids |
| *aox-*JC-R | GCGTAACTTAGGACTTGTG |  |
| *hyg* F | CGACAGATCCGGTCGGCATCTACTCTATTTCTT | Detection of transformants |
| *hyg* R | TCTCGTGCTTTCAGCTTCGATGTAGGAGGG |  |

**Table S2** Reads mapped to the reference *P. ostreatus* CCMSSC00389 genome.

| Sample | Clean reads | Clean bases | Error rate (%) | Q30(%) | GC content (%) | mapped data（reads） |
| --- | --- | --- | --- | --- | --- | --- |
| CK_1 | 51469780 | 7648269405 | 0.0231 | 96.12 | 53.61 | 42588236(82.74%) |
| CK_2 | 50076012 | 7444896626 | 0.0231 | 96.14 | 53.57 | 41529908(82.93%) |
| CK_3 | 54045198 | 8034656676 | 0.0231 | 96.12 | 53.64 | 44756233(82.81%) |
| HS_1 | 49951886 | 7432741108 | 0.0237 | 95.44 | 53.30 | 39854083(79.78%) |
| HS_2 | 44408118 | 6613408411 | 0.0235 | 95.61 | 53.07 | 34459653(77.60%) |
| HS_3 | 40891018 | 6078340360 | 0.0242 | 94.93 | 53.03 | 31593008(77.26%) |
| cPTIO_HS_1 | 43559954 | 6483136561 | 0.0236 | 95.59 | 53.15 | 34550050(79.32%) |
| cPTIO_HS_2 | 44266504 | 6586374560 | 0.0233 | 95.86 | 53.19 | 35216085(79.55%) |
| cPTIO_HS_3 | 51346700 | 7644311860 | 0.0237 | 95.45 | 53.11 | 40735610(79.33%) |
| SNP_HS_1 | 45512286 | 6772757831 | 0.0238 | 95.34 | 53.13 | 35381787(77.74%) |
| SNP_HS_2 | 42291674 | 6298038920 | 0.0237 | 95.46 | 53.33 | 33512372(79.24%) |
| SNP_HS_3 | 49600970 | 7398259841 | 0.0236 | 95.50 | 53.19 | 38911010(78.45%) |


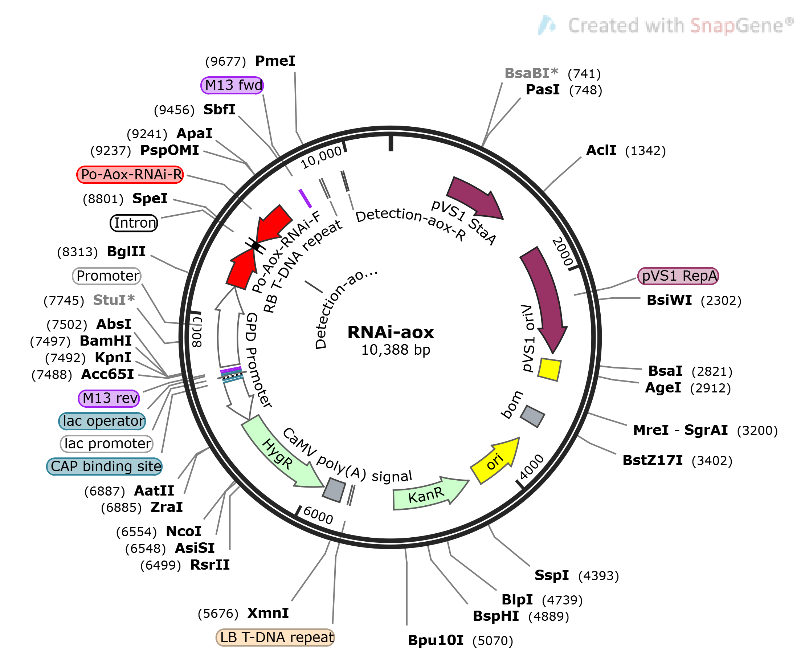


**Fig. S1** Structure of the RNAi-*aox* vector.


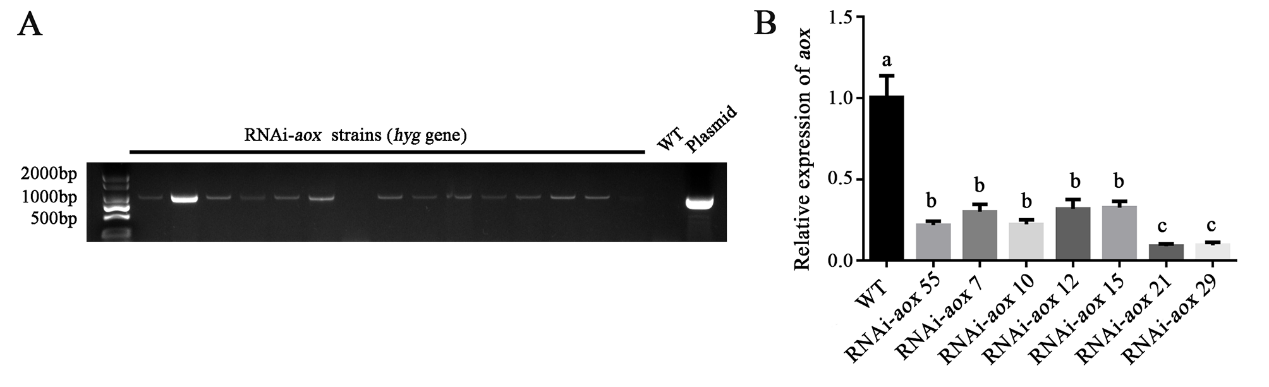


**Fig. S2** Validation and selection of RNAi-*aox* strains. A. Preliminary selection of RNAi-*aox*-transformed strains by amplification of the *hyg* gene. B. qPCR analysis of the expression of *aox* in the tested strains.


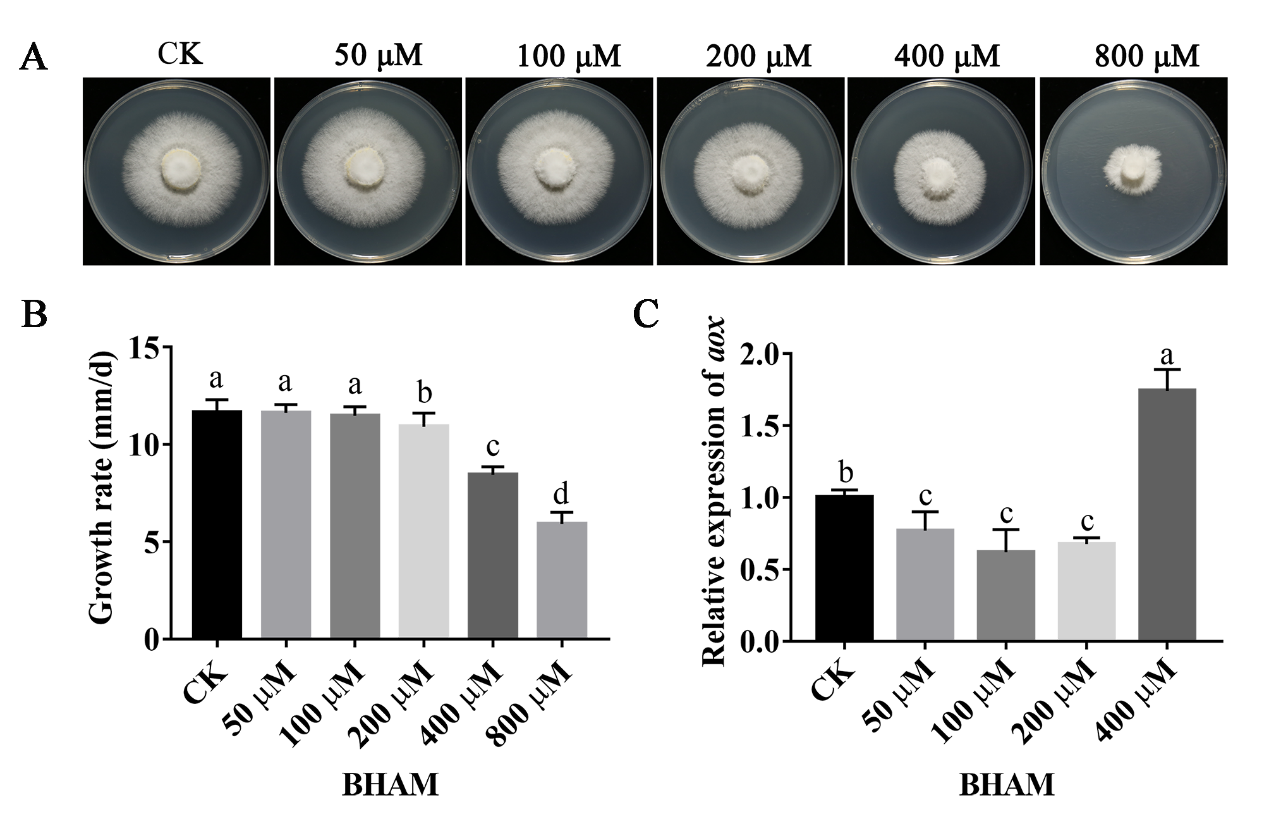


**Fig. S3** Regulation of mycelial growth and *aox* gene expression by different concentrations of BHAM. A. Effects of different concentrations of BHAM on colony morphology. B. Effects of different concentrations of BHAM on the mycelial growth rate. C. Effects of different concentrations of BHAM on *aox* gene expression in mycelia. The values are presented as the means ± SEs from three independent experiments. Different letters indicate signiﬁcant differences among the samples (*P*<0.05 according to Duncan’s test).
